# Recent advances in innovative strategies for plant disease resistance breeding

**DOI:** 10.3389/fpls.2025.1586375

**Published:** 2025-05-21

**Authors:** Dekun Wang, Ruojiao Yang, Mengting Liu, Haolong Li, Haitao Li, Wenya Yuan, Haitao Zhang

**Affiliations:** State Key Laboratory of Biocatalysis and Enzyme Engineering, School of Life Sciences, Hubei University, Wuhan, China

**Keywords:** plant-pathogen interaction, plant immunity, *susceptibility* gene, *resistance* gene, NLR, pathogen effector, gene editing, engineering

## Abstract

Plant disease poses a great threat to crop production. The mechanisms underlying plant-pathogen interactions are critical research topics worldwide. In recent years, significant breakthrough studies have been reported, broadening our understanding of plant immunity. Based on these findings, many strategies have been developed to improve plant defense against various diseases. Here, we summarize these strategies and their applications in studies aimed at promoting crop resistance. Besides domain swapping, gene shuffling, and random mutation, three additional strategies have been developed in the last decade. The first strategy is gene editing of host *susceptibility* (*S*) genes to prevent pathogen infection. Editing of *Mlo* and *DMR6* gene in many species are good examples of this approach. The second strategy is editing the promoters of host *S* genes or *resistance* (*R*) genes. This strategy is widely used to counteract *Xanthomonas*, such as modifying the promoters of *LOB1* and *SWEET* genes in several crops to enhance resistance. The third strategy is designing *R* gene products, especially nucleotide-binding and leucine-rich repeat (NLR) receptors. This approach is based on the growing knowledge of the structural features and mechanisms of NLRs, which have seen significant advances recently. To date, all NLR-engineering attempts have focused on rice paired NLRs, such as Pikp-1/Pikp-2 (allelic to Pikm-1/Pikm-2) and RGA4/RGA5. The bioengineering of these NLRs provides a promising method to combat diverse pathogens. Detailed studies in many crops are also discussed in this review, organized around these strategies. In summary, with progresses in understanding plant immune mechanism, many innovative molecular strategies are available to mitigate the threat of plant pathogens in the future.

## An overview of plant pathology development

Plants are very important to human society. Besides releasing oxygen, they provide food, fruits and even clothing for our survival. In the world today, the population is continually growing and the demand for more crops is also increasing every day, which brings more pressure on crop production. In agriculture, crop yield is threatened by many biotic and abiotic stresses. Various pathogens constitute the main biotic stresses in nature, which have caused significant yield losses throughout history.

To solve these problems, the first step is to understand whether plants can develop resistance to pathogens. In the 1950s, the gene-for-gene hypothesis was proposed by Flor, based on his studies on flax rust. In this model, the resistance occurs when the plant’s *resistance* (*R*) gene and the pathogen’s *avirulence* (*Avr*) gene are both present. This hypothesis demonstrates that plants can genetically resist pathogens and laid the foundation for crop disease resistance breeding and molecular plant pathology. Since the 1990s, hundreds of *Avr* and *R* genes have been cloned from different pathogens and plants, benefitting the improvement of crop breeding.

Based on the accumulation of knowledge about *Avr* and *R* genes, researchers have begun to realize that plants have developed many strategies to overcome the invasion of pathogens during evolution. Unlike vertebrates, plants lack an adaptive immune system and rely entirely on innate immunity to defend against pathogens. This type of immunity is highly evolved and comprises two tiers of response. The first layer of defense takes place on or outside the plant cell membrane, which can respond to pathogen invasion immediately. Usually, pathogens possess small conserved molecules, called pathogen-associated molecular patterns (PAMPs) or microbe-associated molecular patterns (MAMPs), on their surface. Plants utilize membrane-anchored receptors, known as pattern recognition receptors (PRRs), to recognize PAMPs and trigger PAMP-triggered immunity (PTI). The PTI process is effective against a broad range of pathogens. The most well-known PRRs are FLS2 and CERK1, which are receptor-like kinases (RLKs) for bacterial flagellin and fungal chitin respectively (Gomez-Gomez and Boller, 2000; Miya et al., 2007). However, this immunity is relatively weak, and many pathogens can release effectors or other molecules to suppress it. Meanwhile, pathogens can also inject other effectors into plant cells to manipulate host metabolism and gene expressions for their survival and reproduction. This process is called effector-triggered susceptibility (ETS). To counteract ETS, plants evolved a second layer of defense, known as effector-triggered immunity (ETI), which involves the recognition of pathogen effectors by different kinds of *resistance* (*R*) gene products. This model is commonly referred to as the ‘zig-zag’ model (Jones and Dangl, 2006). Pathogen effectors are highly diverse, even among strains of the same species, resulting in ETI specificity. The ETI process usually induces a strong immune response, often accompanied by hypersensitive response (HR) that limits pathogen spread in a very short time. Nonetheless, immutable boundaries do not exist between PTI and ETI, and they are indispensable and function together in nature sometimes (Yuan et al., 2021).

## Plant *R* genes function in diverse ways to defend against pathogens

**Figure 1:**
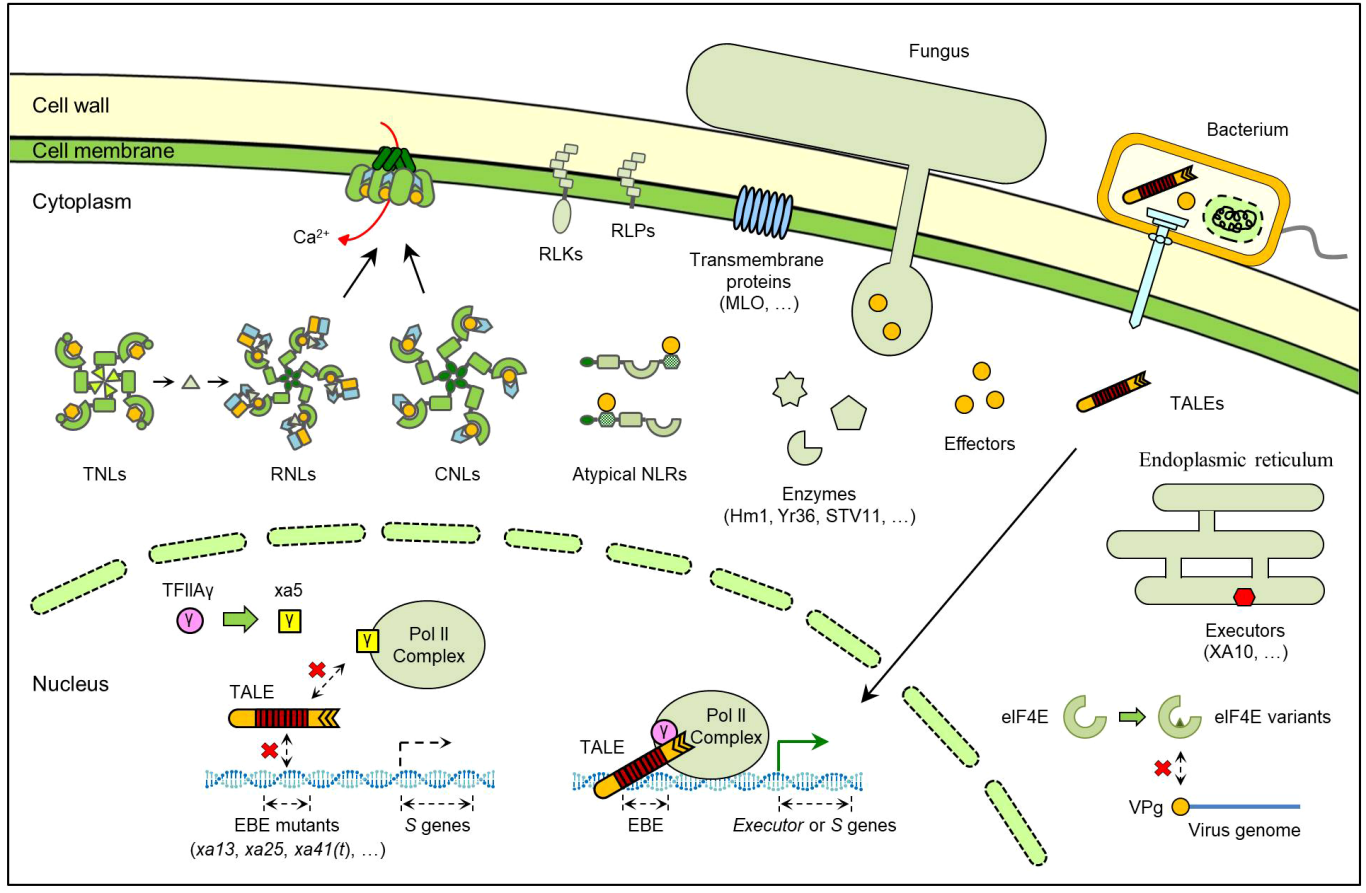
Model of different *R* gene products in plant-pathogen interactions. The products encoded by cloned *R* genes primarily include NLRs, RLKs/RLPs, executors and enzymes. Recessive *R* genes are usually mutations of *S* genes, including protein variants and promoter mutations.

Among all the cloned *R* genes, NLR-encoding genes constitute the largest group and are present in almost all land plants (Gao et al., 2018). Typical NLRs share similar protein structures except for their N-termini, which are categorized into coiled-coil (CC) motif, Toll/Interleukin-1 receptor (TIR) domain and resistance to powdery mildew 8 (RPW8) domain. Based on this, typical NLRs are classified as CC-NLRs (CNLs), TIR-NLRs (TNLs) and RPW8-NLRs (RNLs) (Maruta et al., 2022). Recently, significant progresses have been achieved in NLR protein structure and mechanism studies. It has been revealed that CNLs and TNLs can interact with pathogen effectors directly or indirectly, while RNLs primarily mediate downstream signaling of CNLs and TNLs (Zhang et al., 2022b). All three NLRs classes can be assembled into resistosomes to trigger resistance, inducing Ca^2+^ influx and producing small bioactive molecules (**Figure 1**) (Wang et al., 2019a; Ma et al., 2020; Jacob et al., 2021). These breakthroughs advance our understanding of plant innate immunity and enable applications in breeding practices, such as wheat Sr35 (Zhao et al., 2022).

Meanwhile, many crop NLRs are atypical, which contain additional integrated domains (IDs), including WRKY, kinase, heavy metal-associated (HMA), and zinc-finger BEAF and DREF (zf-BED) domains (Zhang et al., 2022b). In several atypical NLRs, the IDs act as ‘integrated decoys’ to bind pathogen effectors (**Figure 1**) (Jones and Dangl, 2006). However, not all the IDs serve as decoys; for example, the zf-BED domains of rice XA1 and XA14 could not directly interact with pathogen effectors (Zhang et al., 2020; Yoshihisa et al., 2022). Additionally, many atypical NLRs require partner NLRs, usually known as ‘helper NLRs’, to form NLR pairs (also called paired NLRs) and confer resistance (Jones et al., 2016). In rice, many cloned blast *R* genes encode NLR pairs, such as RGA4/RGA5, Pikp-1/Pikp-2 and its allelic genes (Zhang et al., 2022b).

RLKs and receptor-like proteins (RLPs) represent another major class of *R* gene products. Although classified as PRRs in many reports, their discoveries stemmed from the gene-for-gene hypothesis. The examples include Xa21 and Cf-9 (**Figure 1**). *Xa21* is the first cloned *R* gene in rice and confers resistance to bacterial blight (Song et al., 1995). *Cf-9* is the first cloned tomato *R* gene for resistance to the fungus *Cladosporium fulvum* (Jones et al., 1994). Xa21 protein is an RLK containing an extracellular leucine-rich repeat (LRR) domain and an intracellular kinase domain, while Cf-9 is an LRR-RLP lacking the kinase domain (Jones et al., 1994; Song et al., 1995). There are also some other RLKs that contain different kinds of extracellular domains, such as the XA4 and Pi-d2. XA4 is a cell wall-associated kinase (WAK) conferring bacterial blight resistance in rice, and Pi-d2 contains an extracellular B-lectin domain and mediates rice blast resistance (Chen et al., 2006; Hu et al., 2017).

Besides NLRs and RLKs, another interesting class of *R* gene products is the executors. The executor *R* genes harbor special effector binding elements (EBEs) in their promoters and can be induced by transcription activator-like effectors (TALEs) secreted by *Xanthomonas*, which includes many species that can infect large numbers of crops worldwide (**Figure 1**) (Timilsina et al., 2020; Zhang et al., 2022a). The common feature of these executors is that they trigger significant defense responses, even cell death (Zhang et al., 2015). To date, all the reported executor *R* genes originate from pepper and rice. Among them, *Bs3* and *Bs3-E* encode flavin-dependent monooxygenase (FMO) homologs. The others, including *Bs4C-R*, *Xa7*, *Xa10*, *Xa23* and *Xa27*, all encode small special proteins with low similarity to known proteins (Zhang et al., 2015, 2022).

Notably, there are many special recessive *R* genes. These genes can be considered as mutations of *susceptibility* (*S*) genes targeted by pathogen effectors (**Figure 1**) (Timilsina et al., 2020; Zhang et al., 2022a). The well-known examples of this class include barley *mildew resistance locus O* (*mlo*) and rice *xa13* genes. *Mlo* encodes a seven-transmembrane (7-TM) protein that negatively regulates cell death and plant defense (Buschges et al., 1997; Piffanelli et al., 2002). Homozygous *mlo* mutants can confer resistance to powdery mildew fungus in barley (Buschges et al., 1997). The recessive *xa13* results from a mutation in the EBE of *OsSWEET11*, which is targeted by *Xanthomonas oryzae* pv. *oryzae* (*Xoo*) effector PthXo1 (Chu et al., 2006; Yang et al., 2006; Moscou and Bogdanove, 2009). *Xoo* TALEs can also bind to the EBEs of other *SWEET* genes in rice, and mutations in these EBEs have generated other recessive *R* genes, such as *xa25* and *xa41(t)* (Liu et al., 2011; Hutin et al., 2015).

Some recessive *R* genes are alternative forms of the corresponding *S* genes, because they are essential components for gene expression or translation (**Figure 1**). The rice *xa5* gene encodes a protein harboring a V39E amino acid substitution in the gamma subunit of the basal transcription factor IIA 5 (TFIIAγ5) (Iyer and McCouch, 2004; Jiang et al., 2006). In *Xa5*-carrying rice, *Xoo* TALEs hijacks TFIIAγ5 for inducing host gene expression. In contrast, the mutated TFIIAγ5^V39E^ blocks TALE binding and leads to passive resistance (Zhang and Wang, 2013; Yuan et al., 2016). Similar results have been found in cloning of *R* genes against viruses. The potyviruses use a genome-linked viral protein, VPg, to recruit host eukaryotic translation initiation factor 4E (eIF4E) or its isoform eIF(iso)4E for viral replication (Wang and Krishnaswamy, 2012). Coincidentally, many *R* genes mediating resistance to different potyviruse species were finally found to be variants of eIF4E or eIF(iso)4E (Wang and Krishnaswamy, 2012).

Other *R* gene products also exhibit great functional diversity (**Figure 1**). The first cloned plant *R* gene, *Hm1* in maize, encodes an enzyme that detoxifies the *Helminthosporium carbonum* (HC) toxin from *Cochliobolus carbonum* (Johal and Briggs, 1992). Wheat *Yr36* encodes a kinase-START domain protein that phosphorylates other proteins to increase reactive oxygen species (ROS) and suppresses photosynthesis during stripe rust infection (Fu et al., 2009; Wang et al., 2019b). The rice *STV11* gene encodes a sulfotransferase that converts salicylic acid (SA) into sulphonated SA (SSA) during the resistance to rice stripe virus (RSV) (Wang et al., 2014a). Due to space limitations, other cloned plant *R* gene products are not discussed here. Collectively, all these *R* genes provide a foundation for developing new breeding strategies to enhance crop resistance.

## Strategies for improving crop resistance

### Strategy 1. Domain swapping and gene shuffling of *R* genes

**Figure 2:**
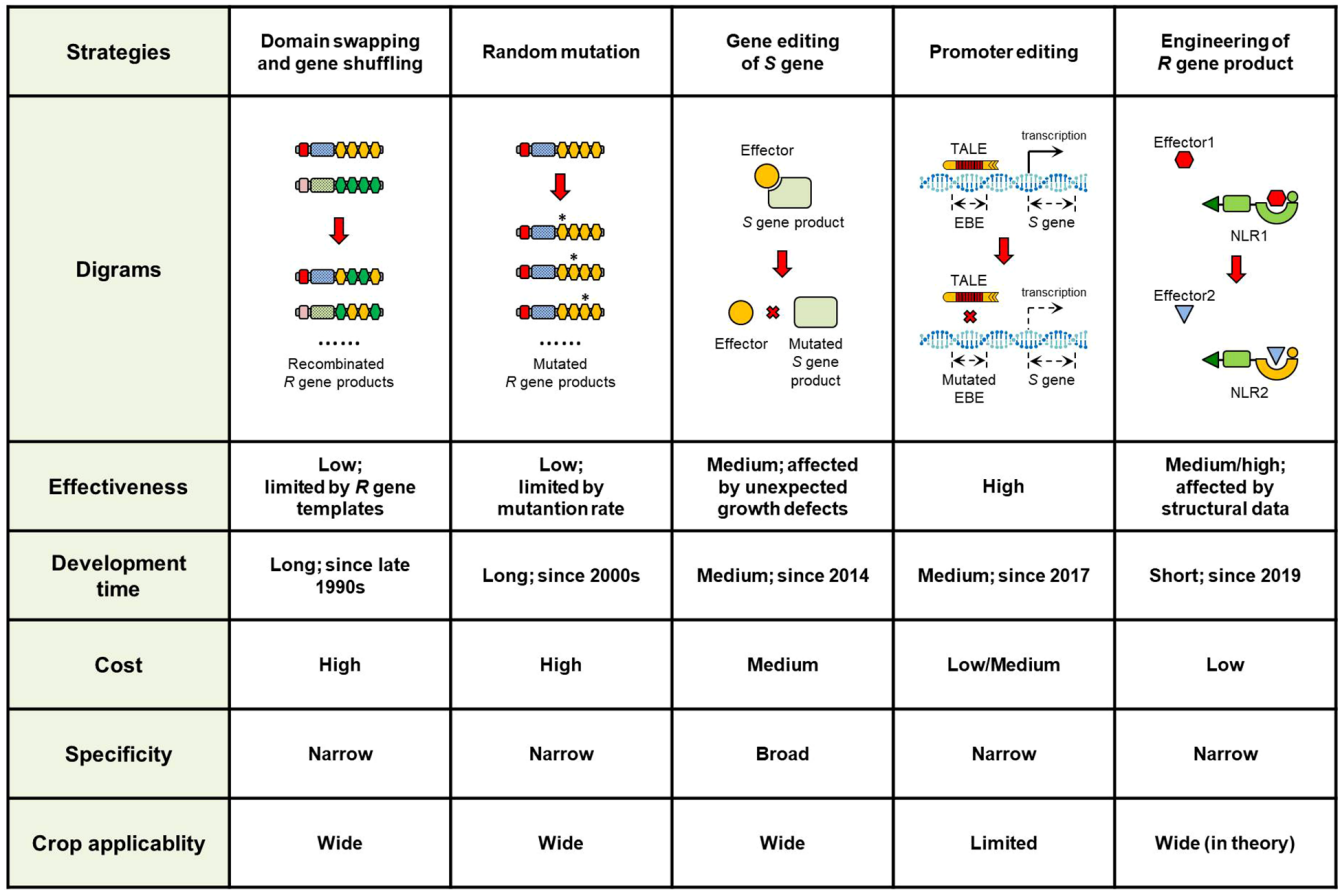
Comparison of different strategies to improve plant disease resistance. The five strategies were compared based on effectiveness, development time, cost, specificity, and crop applicability.

Domain swapping is a technique to construct chimeric genes or proteins formed by exchanging functional domains. Gene shuffling, in contrast, involves generating constructs through PCR amplification using homologous *R* genes as templates. Both methods have long served as the strategic approaches in plant immunity research to identify critical sequences or residues in cloned *R* gene products (**Figure 2**). The earliest application of these methods can be traced back to the studies on flax *L* and tomato *Cf4/9* loci (Ellis et al., 1999; Wulff et al., 2001). The *L* locus harbors multiple allelic NLR genes that confer strain-specific resistance against the flax rust fungus *Melampsora lini* via recognition of distinct effector proteins. Intragenic exchanges among these genes had produced chimeric genes, which revealed the TIR and LRR domains as key determinants of resistance spectrum (Ellis et al., 1999).

These methods have also been used to generate novel *R* genes with enhanced specificity. Using the *L* locus again as an example, *L5* and *L6* are two alleles that confer resistance to different variants of the fungal effector AvrL567. After domain swapping, a chimera protein exhibited recognition of a previously non-recognized AvrL567 variant when expressed in tobacco (Ravensdale et al., 2012).

Practically speaking, the value of these methods lies on discovering the critical functional residues in *R* gene products, rather than exploiting for novel recognition profile. Both approaches require homologous *R* genes as initial templates, making them particularly suitable for NLR- and RLK-encoding genes, as recombination within LRR domains would expands combinatorial possibilities (**Figure 2**). However, the templates would also be the limits of the resistance spectrum potential in most cases, and the new recognition profiles are often limited within the same pathogen species (Zhao et al., 2009; Ravensdale et al., 2012).

### Strategy 2. Random mutation of *R* genes

Mutation is the engine of evolution. As a result, random mutagenesis has emerged as a common strategy to expand *R* gene recognition profiles (**Figure 2**). For instance, *Rx* encodes an NLR that confers resistance to potato virus X (PVX) by recognizing its coat protein, CP-TK. Error-prone PCR was used to mutate the LRR domain of Rx. After screening more than two thousand clones, mutants with broader recognition spectra for CP-KR variants and poplar mosaic virus (PopMV) were identified (Farnham and Baulcombe, 2006). In another mutant library targeting the remaining regions of Rx, several mutants with increased resistance to PopMV were also obtained (Harris et al., 2013). The potato NLR R3a detects *Phytophthora infestans* effector AVR3a but shows limited response to the AVR3a^EM^ variant. By constructing a random mutant library of R3a, researchers successfully isolated multiple variants with enhanced sensitivity to AVR3a^EM^, thereby broadening its resistance spectrum (Segretin et al., 2014).

Similar studies have been reported in other pathosystems to generate artificially evolved *R* genes with expanded recognition specificity by using random mutagenesis (Sueldo et al., 2015). The efficiency varies across different *R* genes. The critical requirement is generating sufficient mutants to ensure adequate coverage of the whole variations (**Figure 2**) (Farnham and Baulcombe, 2006; Segretin et al., 2014; Sueldo et al., 2015). Therefore, the balance between outcome and cost should be considered before performance.

### Strategy 3. Gene editing of *S* genes

In the last decade, gene editing has emerged as one of the most attractive technologies in research. It has developed spans from zinc finger nucleases (ZFNs) and transcription activator-like effector nucleases (TALENs) to clustered regularly interspaced short palindromic repeats (CRISPR)/CRISPR-associated protein (Cas) system (Wang and Doudna, 2023). These technologies enable precise modification of target genes/sequences for specific purposes. Consequently, their applications have rapidly expanded from functional genomic study to crop improvement (Zhang et al., 2022a; Wang and Doudna, 2023). For improving disease resistance, editing crop *S* genes represents a straightforward strategy in practice (**Figure 2**; **Table 1**).

**Table 1:** Plant *S* genes edited for improving disease resistance in recent studies.

*S* gene	Host	Editing tool	Pathogen	Disease name	Reference
*Mlo*	barley	N.A.	*Blumeria graminis* f. sp. *Hordei*	powdery mildew	Buschges et al., 1997
*TaMLO-A1*, *TaMLO-B1*, *TaMLO-D1*	wheat	TALEN, CRISPR/Cas9	*Blumeria graminis* f. sp. *Tritici*	powdery mildew	Wang et al., 2014b; Li et al., 2022b
*CsaMLO1*, *CsaMLO8*, *CsaMLO11*	cucumber	CRISPR/Cas9	*Podosphaera xanthii*	powdery mildew	Tek et al., 2022
*DMR6*	Arabidopsis	N.A.	*Hyaloperonospora parasitica*	downy mildew	van Damme et al., 2008
*StDMR6-1*	potato	CRISPR/Cas9	*Phytophthora infestans*	late blight	Kieu et al., 2021
*SlDMR6-1*	tomato	CRISPR/Cas9	*Pseudomonas syringae* pv. *tomato*	bacterial speck	Thomazella et al., 2021
			*Xanthomonas gardneri*, *Xanthomonas perforans*	bacterial spot	
			*Phytophthora capsici*	phytophthora blight	
			*Pseudoidium neolycopersici*	powdery mildew	
*BoDMR6*	cabbage	CRISPR/Cas9	*Xanthomonas campestris* pv. *campestris*	black rot	Zhang et al., 2025
			*Plasmodiophora brassicae*	clubroot	
*VviDMR6-1*, *VviDMR6-2*	grapevine	CRISPR/Cas9	*Plasmopara viticola*	downy mildew	Giacomelli et al., 2023; Djennane et al., 2024
*MusaDMR6*	banana	CRISPR/Cas9	*Xanthomonascampestris* pv. *musacearum*	*Xanthomonas* wilt	Tripathi et al., 2021
*ObDMR6*	sweet basil	CRISPR/Cas9	*Peronospora belbahrii*	downy mildew	Hasley et al., 2021
*OsS5H1*, *OsS5H2*, *OsS5H3*	rice	CRISPR/Cas9	*Xanthomonas oryzae* pv. *oryzae*	bacterial blight	Liu et al., 2023
			*Magnaporthe oryzae*	blast	
*eIF4E1*	Arabidopsis	CRISPR-Cas9-cytidine deaminase	*Clover yellow vein virus*	/	Bastet et al., 2019
*nCBP-1*, *nCBP-2*	cassava	CRISPR/Cas9	*Cassava brown streak virus*, *Ugandan cassava brown streak virus*	brown streak	Gomez et al., 2019
*CsLOB1*	citrus	CRISPR/Cas9	*Xanthomonas citri* subsp. *citri*	canker	Jia et al., 2017
*TaPsIPK1*	wheat	CRISPR/Cas9	*Puccinia striiformis* f. sp. *Tritici*	wheat stripe rust	Wang et al., 2022
*SlBs5*, *SlBs5L*	tomato	CRISPR/Cas9	*Xanthomonas gardneri*, *Xanthomonas perforans*	bacterial spot	Ortega et al., 2024
*SlPMR4*	tomato	CRISPR/Cas9	*Oidium neolycopersici*	powdery mildew	Santillan Martinez et al., 2020
*SlPMR4*	tomato	CRISPR/Cas9	*Phytophthora infestans*	late blight	Li et al., 2022a
*StDND1*	potato	CRISPR/Cas9	*Phytophthora infestans*	late blight	Kieu et al., 2021
*StCHL1*	potato	CRISPR/Cas9	*Phytophthora infestans*	late blight	
*Pi21*	rice	CRISPR/Cas9	*Magnaporthe oryzae*	blast	Yang et al., 2023 (combined with editing *OsSULTR3;6* promoter); Tao et al., 2021 (combined with editing *Bsr-d1* and *Xa5*)
*Bsr-d1*	rice	CRISPR/Cas9	*Magnaporthe oryzae*	blast	Tao et al., 2021 (combined with editing *Pi21* and *Xa5*)
*TFIIAγ5/Xa5*	rice	CRISPR/Cas9	*Xanthomonas oryzae* pv. *oryzae*	bacterial blight	Tao et al., 2021 (combined with editing *Pi21* and *Bsr-d1*)
*TFIIAγ5/Xa5*	rice	Prime editing	*Xanthomonas oryzae* pv. *oryzae*	bacterial blight	Gupta et al., 2023 (combined with knock-in EBE into *xa23* promoter)
*RBL1*	rice	CRISPR/Cas9	*Xanthomonas oryzae* pv. *oryzae*	bacterial blight	Sha et al., 2023
			*Magnaporthe oryzae*	blast	
			*Ustilaginoidea virens*	false smut	
*ZmNANMT*	maize	CRISPR/Cas9	*Cochliobolus heterostrophus*	southern leaf blight	Li et al., 2023
			*Setosphaeria turcica*	northern leaf blight	
			*Fusarium verticillioides*	*Fusarium* stalk rot	
*BoBPM6*	cabbage	CRISPR/Cas9	*Fusarium oxysporum*	*Fusarium* wilt	Zhang et al., 2025
			*Xanthomonas campestris* pv. *campestris*	black rot	
			*Plasmodiophora brassicae*	clubroot	

The best-characterized example is loss-of-function mutations in the *Mlo* gene. The barley *mlo* mutants have provided durable broad-spectrum resistance against almost all *Blumeria graminis* f.sp. *hordei* (*Bgh*) isolates in agriculture since the late 1970s (Kusch and Panstruga, 2017). The *mlo* gene was isolated via map-based cloning almost three decades ago (Buschges et al., 1997). It encodes a seven-transmembrane protein that is critical for mediating powdery mildew susceptibility. As a calcium-regulated calmodulin-binding protein, MLO protein suppresses host defense reactions including cell wall reinforcement and hypersensitive responses, facilitating pathogen invasion (Piffanelli et al., 2002). Subsequent studies demonstrated that loss function of *Mlo* confers resistance in over 10 plant species, including *Arabidopsis*, wheat, and grapevine (Kusch and Panstruga, 2017). Using different gene editing tools, *Mlo*-edited plants have been generated in wheat and cucumber (**Table 1**). Unlike barley, bread wheat is hexaploid, and only complete loss of all the three *Mlo* homologs, *TaMLO-A1*, *TaMLO-B1* and *TaMLO-D1*, had led to the resistance to *Blumeria graminis* f. sp. *tritici* (*Bgt*) (Wang et al., 2014b; Li et al., 2022b). Although cucumber is a diploid, it has many *Mlo* homologs. Triple mutants of *CsaMLO1*, *CsaMLO8* and *CsaMLO11* were generated by CRISPR/Cas system and exhibited complete resistance to *Podosphaera xanthii* (Tek et al., 2022).

*Downy Mildew Resistant 6* (*DMR6*) is an *S* gene first identified in *Arabidopsis* and then applied in many crops (**Table 1**). It encodes a salicylic acid 5-hydroxylase (S5H) that converts salicylic acid (SA) into 2,5-Dihydroxybenzoic acid (2,5-DHBA) and plays a negative role in plant immunity (Zhang et al., 2017). The *Arabidopsis dmr6* mutants displayed resistance to *Hyaloperonospora parasitica*, the pathogen of downy mildew (DM) (van Damme et al., 2008). The potato genome contains two *DMR6* homologs. CRISPR/Cas9-generated deletion mutants of *StDMR6–1* showed increased resistance to the oomycete pathogen *Phytophthora infestans*, which is the causal agent of late blight (Kieu et al., 2021). Inactivation of *SlDMR6–1* by CRISPR/Cas9 made tomato resistant to a broad spectrum of pathogen, including bacteria, oomycetes, and fungi (Thomazella et al., 2021). In grapevine, CRISPR/Cas9 editing of *VviDMR6–1* and *VviDMR6–2* could reduce the susceptibility to downy mildew (Giacomelli et al., 2023; Djennane et al., 2024). Knocking-out of *BoDMR6* in cabbage resulted in lower disease index of black rot and clubroot (Zhang et al., 2025). Editing *MusaDMR6* in banana and *ObDMR6* in sweet basil enhanced their resistance to *Xanthomonas campestris* pv. *musacearum* (*Xcm*) and *Peronospora belbahrii* respectively (Hasley et al., 2021; Tripathi et al., 2021). In rice, the homologs of *DMR6*, also called *OsS5H1*, *OsS5H2*, and *OsS5H3*, were simultaneously edited by CRISPR/Cas9. The triple mutant exhibited stronger resistance to *Xoo* and *Magnaporthe oryzae* (*M. oryzae*) than any single mutants (Liu et al., 2023).

Other attempts at *S* gene editing have also been carried out (**Table 1**). To combat potyviruses, *eIF4E1*, *nCBP-1* and *nCBP-2* were modified in *Arabidopsis* and cassava (Bastet et al., 2019; Gomez et al., 2019). To overcome citrus canker, the *CsLOB1* gene was mutated using CRISPR/Cas9 technology (Jia et al., 2017). The wheat kinase gene *TaPsIPK1* was recently discovered as an *S* gene, and its inactivation by CRISPR/Cas9 conferred resistance to wheat stripe rust (Wang et al., 2022). In tomato, CRISPR/Cas9-mediated mutagenesis of *SlBs5*, *SlBs5L* and *SlPMR4* increased the resistance to bacterial spot, powdery mildew and late blight (Santillan Martinez et al., 2020; Li et al., 2022a; Ortega et al., 2024). Knocking out *StDND1* and *StCHL1* made potato plants resistant to late blight (Kieu et al., 2021). Editing *Pi21*, *Bsr-d1* and *TFIIAγ5/Xa5* genes mimicked the resistance of *pi21*, *bsr-d1* and *xa5* in rice (Tao et al., 2021; Gupta et al., 2023; Yang et al., 2023). Meanwhile, gene editing of rice *RESISTANCE TO BLAST1* (*RBL1*), maize *ZmNANMT* and cabbage *BoBPM6* could even result in broad-spectrum resistance (Li et al., 2023; Sha et al., 2023; Zhang et al., 2025).

*S* gene inactivation has been attracting increasing attention in studies because of its high effectiveness. However, there are still some considerations to be noted. The most important consideration is that knockout of many *S* genes could lead to unexpected developmental impairments (**Figure 2**). This phenomenon has been observed in multiple crops. To address this, one option is to select alternative *S* genes for editing, while another is to screen large-scale mutant populations for ideal phenotypes. The later approach has proven feasible in wheat and rice. Knockout of *Mlo* homologs in wheat caused reduced plant height and grain yield. Through screening additional *mlo* mutants, the *Tamlo-R32* mutant without growth penalties was recently identified (Li et al., 2022b). The *rbl1* mutant exhibited many growth defects in rice, whereas the *rbl^Δ12^* mutant, generated via multiplex gene editing, achieved a balance between growth and resistance (Sha et al., 2023).

The success in *S* gene inactivation-mediated resistance has extended its application to studies on resistance negative regulators. Some negative regulator genes even function similarly to *S* genes in plant-microbe interactions. For example, the *GbWAKL14* gene negatively regulated defense response by modulating reactive oxygen species (ROS) levels, and its CRISPR/Cas9-mediated inactivation enhanced the wilt disease resistance in cotton (Zhao et al., 2024). Although their mechanistic contributions to pathogen invasion may vary, these genes provide valuable resources for improving crop resistance. Given the limited availability of cloned *R* and *S* genes in certain crops, modification of negative regulators offers a viable short-term alternative option.

### Strategy 4. Promoter editing for resistance

Besides editing the coding sequences of crop *S* genes, promoter modification is another strategy for improving resistance, especially when combating *Xanthomonas*. Many *Xanthomonas* species exploit TALEs to induce the host *S* genes to acquire nutrients and induce disease symptoms. As a result, artificially editing EBEs in *S* gene promoters will attenuate their expression activation, mimicking the function of some recessive *R* genes (**Figure 2**; **Table 2**). In citrus, the *CsLOB1* gene is induced by *Xanthomonas citri* subsp. *citri* (*Xcc*) effector PthA4. It has been reported in many studies that editing the PthA4 EBE in *CsLOB1* promoter significantly reduced or even eliminated the canker symptoms (Jia et al., 2017, 2019; Jia and Wang, 2020; Jia et al., 2022, 2024). *Xanthomonas axonopodis* pv. *manihotis* (*Xam*), also called *Xanthomonas phaseoli* pv. *manihotis* (*Xpm*), causes bacterial blight in cassava. Its major virulence effector, TAL20, upregulates the expression of *MeSWEET10a* during infection. Editing the TAL20 EBE in *MeSWEET10a* promoter resulted in bacterial blight resistance in cassava (Elliott et al., 2024; Wang et al., 2024c). Moreover, using a DMS3-ZF system, which fused the artificial zinc-fingers (ZFs) to a DNA methylation-related protein, the methylation within and around the EBE_TAL20_ was increased. This modification prevented the transcription activation of *MeSWEET10a* and decreased the disease symptoms in cassava (Veley et al., 2023). Bacterial blight and bacterial leaf streak disease in rice are both caused by *Xanthomonas*, and several rice *S* genes targeted by TALEs have been identified until now. *OsSWEET11* (also called *Xa13* or *Os8N3*), *OsSWEET13*, and *OsHXK5* are activated by *Xoo* effectors PthXo1, PthXo2, and Tal10a, respectively; *OsSWEET14* is induced by four *Xoo* TALEs, PthXo3, AvrXa7, TalC, and TalF, which bind distinct EBEs; The *OsSULTR3;6* gene is targeted by *Xanthomonas oryzae* pv. *oryzicola* (*Xoc*) effectors Tal2g and Tal5d (Zhang et al., 2022a). As a result, editing the EBEs in these genes led to resistance in rice, and different combinations of these edits had shown broader resistance spectra in multiple studies (Eom et al., 2019; Kim et al., 2019; Li et al., 2019; Oliva et al., 2019; Xu et al., 2019; Li et al., 2020; Ni et al., 2021; Xu et al., 2021; Schepler-Luu et al., 2023; Yang et al., 2023; Gupta et al., 2024; Liu et al., 2024; Wang et al., 2024a).

**Table 2:** Types of promoter editing to enhance plant disease resistance in recent studies.

Type	*S* gene(s)	Host	Editing tool	Edited cis-element	Corresponding effector	Pathogen	Disease name	Reference
1	*CsLOB1*	citrus	CRISPR/Cas9, CRISPR-Cas12a, CRISPR-SpCas9p, LbCas12a-D156R, Cas12a/CBE	EBE_PthA4_ -LOBP	PthA4	*Xanthomonas citri* subsp. *citri* (*Xcc*)	canker	Jia et al., 2017, 2019; Jia and Wang, 2020; Jia et al., 2022, 2024
2	*MeSWEET10a*	cassava	DMS3-ZF, CRISPR/Cas9	EBE_TAL20_	TAL20	*Xanthomonas axonopodis* pv. *manihotis* (*Xam*)	bacterial blight	Veley et al., 2023; Elliott et al., 2024; Wang et al., 2024c
3	*OsSWEET11, OsSWEET14*	rice	CRISPR/Cas9	EBE_PthXo1_, EBE_PthXo3_, EBE_AvrXa7_	PthXo1, PthXo3, AvrXa7	*Xanthomonas oryzae* pv. *oryzae* (*Xoo*)	bacterial blight	Ni et al., 2021
	*OsSULTR3;6*	rice	CRISPR/Cas9	EBE_Tal2g_	Tal2g	*Xanthomonas oryzae* pv. *oryzicola* (*Xoc*)	bacterial leaf streak	
4	*OsSULTR3;6*	rice	CRISPR/Cas9	EBE_Tal2g/Tal5d_	Tal2g/Tal5d	*Xanthomonas oryzae* pv. *oryzicola* (*Xoc*)	bacterial leaf streak	Xu et al., 2021; Yang et al., 2023 (combined with editing *Pi21*)
5	* OsHXK5; OsSULTR3;6*	rice	CRISPR/Cas9	EBE_Tal10a_, EBE_Tal2g_	Tal10a, Tal2g	*Xanthomonas oryzae* pv. *oryzicola* (*Xoc*)	bacterial leaf streak	Wang et al., 2024a
6	*OsSWEET11(Xa13/Os8N3)*	rice	CRISPR/Cas9; Prime editing	EBE_PthXo1_	PthXo1	*Xanthomonas oryzae* pv. *oryzae* (*Xoo*)	bacterial blight	Kim et al., 2019; Li et al., 2019 (combined with editing *TMS5* and *Pi21*); Li et al., 2020; Gupta et al., 2024 (combined with other editings)
7	*OsSWEET11, OsSWEET13, OsSWEET14*	rice	CRISPR-Cas9/Cpf1	EBE_PthXo1_, EBE_PthXo2_, EBE_TalC_, EBE_TalF_, EBE_PthXo3_, EBE_AvrXa7_	PthXo1, PthXo2, TalC, TalF, PthXo3, AvrXa7	*Xanthomonas oryzae* pv. *oryzae* (*Xoo*)	bacterial blight	Eom et al., 2019; Oliva et al., 2019; Xu et al., 2019; Schepler-Luu et al., 2023; Liu et al., 2024
8	*xa23*	rice	CRISPR/Cas9	knock-in EBE_AvrXa23_	AvrXa23	*Xanthomonas oryzae* pv. *oryzae* (*Xoo*)	bacterial blight	Wei et al., 2021
			Prime editing	knock-in EBE_PthXo1_	PthXo1	*Xanthomonas oryzae* pv. *oryzae* (*Xoo*)	bacterial blight	Gupta et al., 2023 (combined with editing *TFIIAγ5*)
			CRISPR/Cas9	knock-in 10 EBEs	PthXo1, PthXo3, AvrXa23, Tal9aBLS256, etc.	*Xanthomonas oryzae* pv. *oryzae* (*Xoo*), *Xanthomonas oryzae *pv. *oryzicola* (*Xoc*)	bacterial blight, bacterial leaf streak	Wang et al., 2024b

After long-term evolution, some crops have developed ‘imitative’ EBEs for TALEs in the promoters of executor-encoding genes to trigger defense responses (Zhang et al., 2022a). Using gene editing tools, promoters of the recessive alleles of executor-encoding genes could be modified to respond to pathogen invasion. Such attempts are mainly focused on rice *xa23*, which encodes the same executor as *Xa23* but lacks the EBE_AvrXa23_ in its promoter (**Table 2**). Using the CRISPR/Cas9 system, EBE_AvrXa23_ could be knocked into the *xa23* promoter with very low probability, rendering rice plants resistant to *Xoo* (Wei et al., 2021). In another study, EBE_PthXo1_ was knocked into the *xa23* promoter to broaden the resistance spectrum of *TFIIAγ5*-edited plants (Gupta et al., 2023). Even an artificial EBE array, which consisted of different EBEs for TALEs of *Xoo* and *Xoc*, could be knocked into the promoter of *xa23*, conferring broad-spectrum resistance in rice (Wang et al., 2024b).

To date, promoter modification of *non-R* or *non-S* genes for resistance enhancement remains unreported. This strategy is also theoretically feasible for genes triggering cell death and defense responses. However, the cis-elements specially responding to pathogens are not always clear in many cases, which would limit the applicability of this approach (**Figure 2**).

### Strategy 5. Engineering of *R* gene products

In recent years, rapid developments have taken place in structural biology. These advancements has provided accumulating details on interactions between *R* gene products and pathogen effectors, enabling the engineering of novel *R* gene products. Such pioneering attempts and studies mainly focused on rice blast resistance (**Figure 2**; **Table 3**). The allelic *R* genes *Pikp* and *Pikm* are each composed of two tandem NLR genes. Both Pikp-1 and Pikm-1 contain a Heavy Metal Associated (HMA) domain that interacts with effectors from *M. oryzae*. Based on structural insights into protein interactions, specific residues from the Pikm-1 HMA domain were swapped into Pikp-1. And one mutant, Pikp-1^NK-KE^, exhibited altered binding to AVR-PikA and AVR-PikE, effectors recognized by Pikm but not Pikp (De la Concepcion et al., 2019). Similar engineering was applied to RGA5, another NLR containing an HMA domain at the carboxyl terminus. Key residues in RGA5 were replaced with Pikp-1-derived AVR-PikD-interacting residues. The engineered NLR, RGA5m1m2, retained the recognition of AVR-Pia and AVR1-CO39 while gaining additional AVR-PikD specificity (Cesari et al., 2022). However, these engineered NLRs conferred resistance only in tobacco. Transgenic rice plants expressing these constructs failed to resist blast disease, possibly due to insufficient structural data on full-length NLR proteins at the time (De la Concepcion et al., 2019; Cesari et al., 2022).

**Table 3:** Recent studies on engineering of NLRs.

Case	NLR	Engineered form	Changement	Plant	Result	Reference
1	Pikp-1	Pikp-1^NK-KE^	mutaion at HMA (based on Pikm-1)	tobacco	obtained additional recognition profile of Pikm (in tobacco)	De la Concepcion et al., 2019
2	RGA5	RGA5^HMA2^	mutaion at HMA (structure prediction)	tobacco, rice	recognized AVR-Pib not AVR-Pia	Liu et al., 2021
3	RGA5	RGA5m1m2	mutaion at HMA (based on Pikm-1)	tobacco	obtained additional recognition profile of Pikm (in tobacco)	Cesari et al., 2022
4	Pikp-1	Pikp-1^OsHIPP19mbl7^	mutaion at HMA (based on OsHIPP19)	tobacco, rice	obtained additional recognition to AVR-PikC and AVR-PikF	Maidment et al., 2023
		Pikp-1^SNK-EKE^	mutaion at HMA (structure-guided)			
5	Pikm-1	Pikm-1^Nano^	replacement of HMA with nanobody	tobacco	obtained the recognition profile of nanobody (in tobacco)	Kourelis et al., 2023
6	RGA5	RGA5^HMA5^	mutaion at HMA (structure based)	tobacco, rice	recognized AVR-PikD not AVR-Pia	Zhang et al., 2024
7	RGA5	RGA5^HMA120^	mutaion at HMA (based on HMA120)	tobacco, rice	recognized AVR-Pita not AVR-Pia	Zhu et al., 2025

With advancements in structural analysis and prediction techniques, engineering NLRs has become feasible in practice (**Table 3**). In a study on RGA5, mutations were introduced into its HMA domain based on structure predictions to interact with a non-cognate effector, AvrPib. The engineered NLR, RGA5^HMA2^, successfully shifted recognition from AvrPia to AvrPib in both tobacco and rice (Liu et al., 2021). A similar outcome was observed with another designed NLR, RGA5^HMA5^, which altered resistance specificity from AVR-Pia to AVR-PikD in rice (Zhang et al., 2024). Through large-scale screening of rice HMA domains, HMA120 was identified as an AVR-Pita-interacting domain. After integrating HMA120 into RGA5, the chimeric RGA^HMA120^ could confer resistance to AVR-Pita-carrying pathogen in rice (Zhu et al., 2025). Likewise, the HMA domain of OsHIPP19 was able to interact with AVR-PikC and AVR-PikF with high affinity. Using structural modeling, two engineered NLRs, Pikp-1^OsHIPP19-mbl7^ and Pikp-1^SNK-EKE^, were generated. Both of them mediated additional resistance to *M. oryzae* strains expressing AVR-PikC and AVR-PikF in rice (Maidment et al., 2023).

The most groundbreaking advance in *R* gene product engineering is the fusion of NLRs with nanobodies (**Table 3**). The nanobodies are small fragments of camelid antibodies and are widely used in biotechnology. Replacing the HMA domain with nanobodies specific to fluorescent proteins (FPs), Pikm-1^Nano^ and Pikm-2 formed a ‘Pikobody’ that conferred resistance against *Potato virus X* (PVX) variants expressing FPs in transgenic tobacco (Kourelis et al., 2023). The Pikobody established an artificial adaptive immunity-like system, enabling plants to combat diverse pathogens, including emerging strains, with potential applications in future crop production. However, it should be noticed that no effector-targeted Pikobody has been engineered to date. As a consequence, more practice is needed to further optimize the system to enhance its practicality.

## Future perspectives

Over three decades of researches have significantly advanced our understanding of plant defense mechanisms against pathogens. Although there are still many unknowns, current insights already provide actionable guidance for crop resistance breeding. Pathogen invasion relies on multiple steps, many of which involve hijacking and exploiting proteins or other molecules in host cells for paying the minimum cost. Conversely, perturbations in pathogen invasion processes can disrupt pathogen viability and lead to infection failure. Such dynamics underpin the evolutionary emergence of recessive resistance genes in plants. As a result, targeted intervention in critical molecular nodes of pathogen-host interplay will still be a common strategy in the future. These nodes include both *S* genes and resistance-negative regulator-encoding genes. More studies on the editing of both the coding regions and promoters of these genes will appear, especially in combination with editing of other functional genes.

With rapid developments in synthetic biology, attempts to engineer specialized NLRs have already appeared. Although these attempts have not reached the point where it can be applied in crop production now, the prospect is broad and attractive. The ‘Pikobody’ system has shown us the possibility that artificial immune receptor proteins could be a general solution in dealing with diverse pathogen threats. Nonetheless, more work should be done to optimize it to meet the needs of different crops. Additionally, expanding the spectrum of engineered receptors will be a key direction in the near future. This could be achieved by targeting PAMPs or other common features of distinct pathogens, as well as by combining recognition regions from multiple receptors. Moreover, synthetic promoters will be another focus. With the help of pathogen-responsive *cis*-elements, many genes could be used to improve crop disease resistance. Currently, such attempts have been made, such as the ‘promoter trap’ in several plants to resist *Xanthomonas* (Zhang et al., 2022a). In the future, more sophisticated promoters will be engineered to combat multiple pathogens.

Artificial intelligence (AI) has emerged as a transformative force in life sciences, redefining many research paradigms. Last year, the Nobel Prize was awarded to three scientists for their contribution to computational protein design and protein structure prediction. Both techniques can be applied in research on plant-pathogen interaction and crop disease resistance. To date, many direct interactions between plant *R* gene products and pathogen effectors remain unclear. And this challenge will soon be addressed with the aid of platforms like AlphaFold. Not only can AI assist in prediction, but structure-based *de novo* design of plant *R* gene products will also be possible in the near future. Recently, artificial antibodies have been successfully designed through a fully *de nove* method and have bound specific epitopes as expected (Bennett et al., 2025). This approach will facilitate the engineering of plant immune receptors. In addition, machine learning will play a key role in identifying essential genes in the response to pathogens based on multi-omics data and in optimizing CRISPR targets to increase editing efficiency.

Ecological factors and impacts should be considered in disease resistance breeding. Plants are colonized by communities of microbes in nature, which include not only pathogens but also beneficial microbes. Some of them can protect plants from being infected by various pathogens. If the plants are engineered to facilitate their colonization, the disease resistance will be enhanced (Ge and Wang, 2025). Meanwhile, plants and pathogens are co-evolving with each other. Therefore, the variation and evolution of pathogens should be considered when engineered *R* genes are applied in breeding. Monitoring the effector diversity among different strains from various locations would provide important information for adapting engineering strategies.

The strategies discussed here largely rely on transgenic process. The public’s attitude towards genetically modified organisms (GMOs) is worth noting. Most of the concerns about GMOs stem from misunderstandings. Communication and transparent policies would be useful to change public perception. Scientists should make more efforts to demonstrate to the public that using gene editing tools can now minimize changes to crop genomes; policymakers should let the public know that all the GMOs are regulated by a series of policies and the GMOs are optional, not mandatory, in daily life. Open-access databases documenting the safety and efficacy of edited traits will also be helpful for the public. In China, the acceptance of GMOs has improved compared with ten years ago. With continuous efforts, disease resistance breeding paradigms will be reshaped in the future.
